# Urban and Rural Differences of Acute Cardiovascular Disease Events: A Study from the Population-Based Real-Time Surveillance System in Zhejiang, China in 2012

**DOI:** 10.1371/journal.pone.0165647

**Published:** 2016-11-01

**Authors:** Weiwei Gong, Xiaolin Wei, Yujia Liang, Guanyang Zou, Ruying Hu, Simin Deng, Zhitong Zhang, Jing Pan, Bernard C. K. Choi, Min Yu

**Affiliations:** 1 Zhejiang Centre for Disease Control and Prevention, Hangzhou, Zhejiang, China; 2 Division of Clinical Public Health and Institute of Health Policy, Management and Evaluation, Dalla Lana School of Public Health, University of Toronto, Toronto, Ontario, Canada; 3 China Global Health Research and Development, Shenzhen, China; Old Dominion University, UNITED STATES

## Abstract

Zhejiang province, China, has implemented a population based, real-time surveillance system that tracks acute cardiovascular diseases (CVDs) events since 2001. This study aimed to describe the system and report CVD incidence, mortality and case-fatality between urban and rural areas in Zhejiang in 2012. The surveillance system employs a stratified random sampling method covering all permanent residents of 30 counties/districts in Zhejiang. Acute CVD events such as coronary heart disease (CHD) and stroke were defined, registered and reviewed based on the adapted MONICA (Monitoring Trends and Determinants in Cardiovascular Disease) definitions. Data were collected from health facilities, vital registries, supplementary surveys, and additional investigations, and were checked for data quality before input in the system. We calculated the rates and compared them by gender, age and region. In 2012, the incidence, mortality and case-fatality of total acute CVD events were 367.0 (CHD 59.1, stroke 307.9), 127.1 (CHD 43.3, stroke 83.8) per 100,000 and 34.6% (CHD 73.2%, stroke 27.2%), respectively. Compared with rural areas, urban areas reported higher incidence and mortality but lower case-fatality rates for CHD (P<0.001), while lower incidence but higher mortality and case-fatality rates for stroke (P<0.001). We found significant differences on CHD and stroke epidemics between urban and rural areas in Zhejiang. Special attentions need to be given to stroke control, especially in rural areas.

## Introduction

Cardiovascular diseases (CVDs), mainly consisting of coronary heart disease (CHD) and stroke, are the number one killer in China [1]. While most western countries have witnessed a gradual decline of the CVD epidemic in the past decades [2–4], China has facing an unprecedented increase since 1990 due to its rapid economic development and associated changes in lifestyles [1, 5, 6]. By 2008, the prevalence rates of CHD and stroke in China had reached 77 and 97 per 10,000, respectively [7]. It is estimated that China will have an additional 21 million CVD cases by 2030 [8]. There is an urgent need to establish a population based real-time surveillance system that enables a systematic and continuous collection of data on CVD cases to monitor the progress of the epidemic [9]. A well-functioning surveillance system also provides evidence-based information to health policy-makers [10]. However, developing countries normally lack the resources and capacity to implement such a sophisticated system for CVD surveillance [11]. The Monitoring Trends and Determinants in Cardiovascular Disease (MONICA) project was established by the World Health Organization (WHO) in 1980s. But it only covered a small proportion of the population in developing countries. China set up the National Stroke Registry in 2007 [12] and subsequently the Acute Myocardial Infarction Registry in 2013 [13]. However, both systems were hospital-based, only tracked cases for a short period, and covered a relatively small population.

Zhejiang is a populous and relatively affluent province located in the eastern China. The province has set up an Internet-based Comprehensive Chronic Disease Surveillance System (ICDSS) in 2001 for non-communicable diseases, including CVDs. The ICDSS covers a representative sample of 16.7 million residents (or 35% of the total population) in the province [14]. It operates based on an adapted framework of the MONICA project. The surveillance system creates a pool that linking databases from different sources to comprehensively investigate the epidemic of acute CVD events in the province. Using data collected from ICDSS, the study aimed to investigate the incidence, mortality and case-fatality of acute CVD events in Zhejiang in 2012, and in particular to examine the urban-rural differences.

## Materials and Methods

### Research Setting

In 2012, Zhejiang had 47 million permanent residents. It had a per capita gross domestic product (GDP) of US$10,100, which was above China’s national average of US$6,100. Life expectancy in Zhejiang was 77.6 years. CVD was the leading cause of death, accounting for 25.3% of total mortalities in 2012 [15].

### Sampling

**Fig 1:**
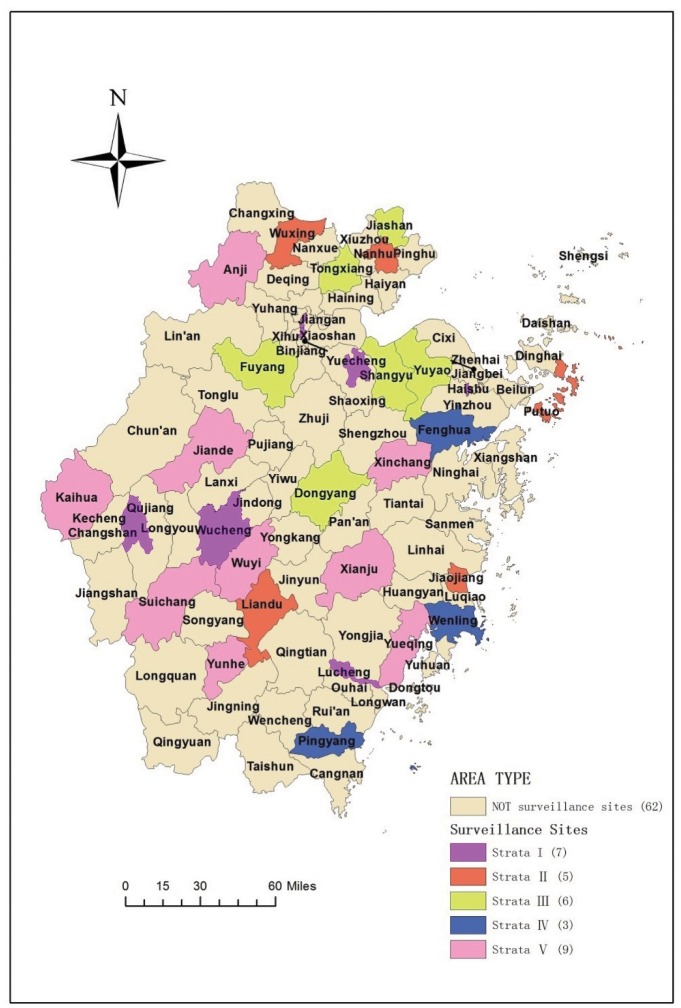
Geographical distribution of the 30 surveillance sites in Zhejiang province, China.

A stratified random sampling method was employed in the ICDSS covering 30 of the 90 counties/districts in Zhejiang [14]. First, five clusters were constructed through K-means clustering based on component scores of social development in a principal component analysis, which categorized all of Zhejiang’s 63 rural counties in three clusters and its 28 urban districts in two clusters. A sample size of 30 counties was estimated to give a standard error of 1% with a coefficient of variation of 5%. Second, 18 rural counties and 12 urban districts were randomly selected from the five clusters as surveillance sites [14]. The clusters distributed evenly across the province (Fig 1). All permanent residents in the selected counties or districts were included in the surveillance system.

### Acute CVD events registration and data quality assurance

In general, the surveillance system collected acute CVD events from three sources: 1) health care facility reports; 2) death certificate reviews; and 3) supplementary surveys. The majority of events were reported by clinical doctors from healthcare facilities, including hospitals and primary care facilities. Hospital-reported cases were identified through a combination of ‘hot pursuit’ (prospective follow-up on admission) and ‘cold pursuit’ (retrospective record analysis after discharge). Primary care doctors based in community health centres or township hospitals also reported cases identified during their consultations with patients. Staff in the Centres for Disease Control and Prevention (CDCs) reviewed death certificates to identify any underreported fatal acute CVD events. In addition, additional investigations were conducted during the annual health examinations that are provided freely to elderly residents in primary care facilities [16].

**Fig 2:**
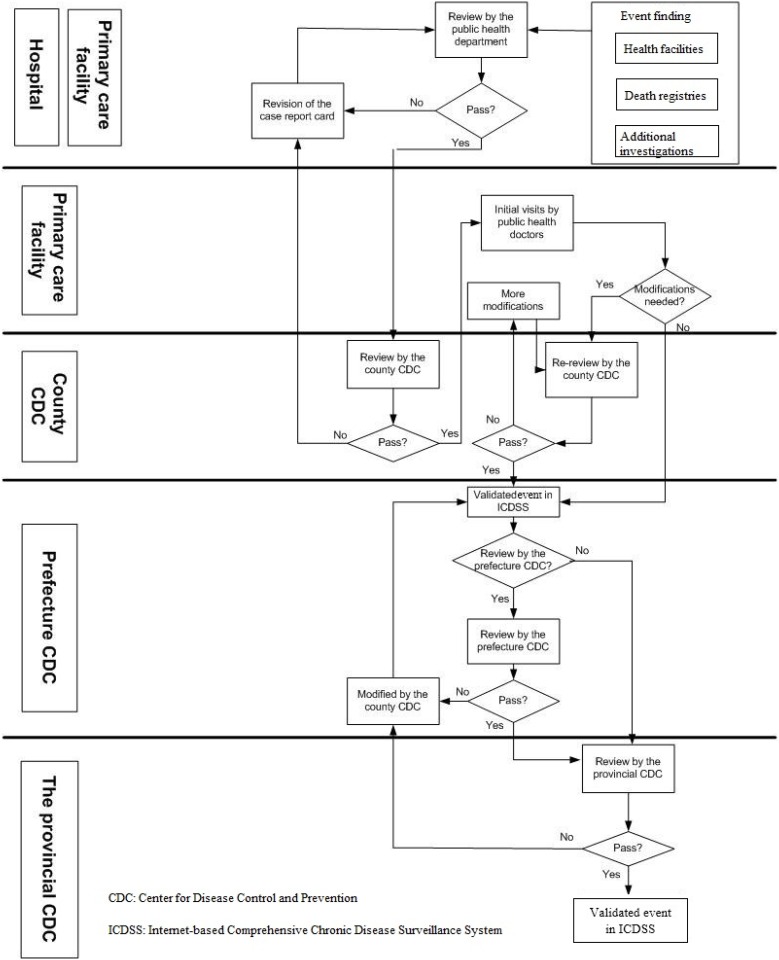
Flowchart of the Internet-based Comprehensive Chronic Disease Surveillance System (ICDSS) in Zhejiang, China.

A strict registration and validation procedure was undertaken once an acute CVD event was detected. First, the public health department of the health care facility reviewed CVD reports submitted by clinical doctors before registering them in the system. Second, all reported new acute CVD events were reviewed and verified by staff in the CDCs from the county to the provincial level. Only when a reported event met the standards, would the CDC staff verify, register and refer it to the next level. After an acute CVD event was registered at the county level, the primary care facility, whose catchment area covers the case, was responsible to validate the event through home visits. Regular training on ICDSS operational guidelines was provided to health care workers for supervision of acute CVD events reported in their facilities. Fig 2 illustrates how the system operates.

### Diagnostic categories and registration

Adapted MONICA definitions of acute CVD events were employed to categorize subtypes of CHD and stroke in ICDSS. CHD consists of three subtypes: 1) acute myocardial infarction (AMI, code I21 I22 in the 10th revision of the International Statistical Classification of Diseases and Related Health Problems [ICD-10], as in MONICA diagnostic category 1 and 2); 2) ischaemic cardiac arrest (ICD code I46, MONICA categories 1, 2 and 3); and 3) unclassifiable deaths (ICD code I24/I25, MONICA category 9) [17]. As MONICA standards for stroke diagnosis offer little pathological information, stroke events in this study were classified according to ICD-10 codes: haemorrhagic stroke (consisting of subarachnoid haemorrhage [I60] and intracerebral haemorrhage [I61]), ischaemic stroke/infarction (I63, consisting of thrombosis and embolism) and unclassifiable stroke (ICD code I64, MONICA category 9) [18]. In general, this classification scheme was consistent with MONICA definitions as both haemorrhagic and ischaemic stroke fell under the MONICA category of ‘definite stroke’. Other demographic information (name, gender, date of birth, and residency status), diagnostic results (including electrocardiogram, computerized tomography, magnetic resonance imaging, coronary angiography, and etc.) and medical history (including hypertension, diabetes, hyperlipidemia, and etc.) were also recorded.

### Data collection and statistical analysis

Data were extracted from the ICDSS server in the Zhejiang Center for Disease Control and Prevention. We conducted the surveillance of CHD and stroke by calculating their incidence, mortality and case-fatality rates. Incidence is defined as the number of new disease events, both first and recurrent, that occur within a year per 100,000 population at risk of developing the disease. Mortality is defined as the number of all fatal acute events within a year per 100,000 population. Case-fatality is the proportion of fatal events within 28 days of onset in all CVD incident events, including both first and recurrent ones [19]. We employed direct age-standardization in estimating incidence, mortality and case-fatality rates. ICDSS did not collect other population information regarding ethnicity, education and household income, so we were not able to adjust for these factors. To ensure comparability of our estimates, the WHO standard population (2001–2025) [20] was adopted as the reference population. We calculated both crude and standardized rates by age, gender and areas, and compared both crude and standardized rates using SAS 9.4 (NC, USA). The main analysis was to examine the rural-urban difference of CVD rates in terms of gender and age.

### Ethic statement

Ethical approval was obtained from the Ethics Committee of the Zhejiang Centre for Disease Control and Prevention. Written informed consent was not obtained from patients or patient relatives. All patient information was anonymized prior to analysis. Access to the ICDSS database was approved by Zhejiang Centre for Disease Control and Prevention.

## Results

A total of 16.7 million people (35% of the total population in the province) were monitored in the surveillance sites. The population under surveillance had similar demographic distributions to the provincial population (Table 1). The distribution of gender, residence and age were similar (p>0.05) in the surveillance population compared with provincial or national population.

**Table 1:** Populations (in millions) and percentage distribution by demographic characteristics in the surveillance sites, compared with two reference populations Zhejiang province and China, 2012.

	Surveillance Sites	Zhejiang	China
Gender^a^			
Male	8.5 (50.9%)	24.0 (50.7%)	693.2 (51.2%)
Female	8.2 (49.1%)	23.3 (49.3%)	660.8 (48.8%)
Residence ^a^§			
Urban	5.9 (35.3%)	15.5 (32.8%)	403.2 (29.8%)
Rural	10.8 (64.7%)	31.8 (67.2%)	950.8 (70.2%)
Age groups^a^			
<35 years	7.1 (42.5%)	19.0 (40.2%)	643.1 (47.5%)
35–64	7.7 (46.1%)	23.6 (49.9%)	583.6 (43.1%)
> = 65 years	1.9 (11.4%)	4.7 (9.9%)	127.3 (9.40%)
Total	16.7 (100.0%)	47.3 (100.0%)	1354 (100.0%)

^a^ Pearson chi-square test was used to compare the population of surveillance sites with the total population of Zhejiang, and China respectively regarding gender, residence status and age. No significant difference was found (p>0.05).

^§^Only participants living in the urban districts of prefectures were registered as urban residents; participants living in rural counties, towns and villages were registered as rural residents.

In 2012, 61,279 acute CVD events were reported, including 9,866 (16%) CHD events and 51,413 (84%) stroke events (Table 2). Acute myocardial infarction, accounting for 56.8% of CHDs, and ischaemic stroke, accounting for 74.2% of strokes, were the two dominant subtypes. In addition, acute myocardial infarction was the most commonly reported CHD among all age groups, while the proportion of ischamemic stroke was higher than haemorrhagic stroke among those older than 35 years.

**Table 2:** Total number and proportion (%) of acute cardiovascular disease subtypes by gender, residence and age groups in Zhejiang, China, 2012.

	Acute cardiovascular diseases	Coronary heart disease				Stroke			
	Total number of acute cardiovascular diseases	Total number of coronary heart disease	Acute myocardial infarction (%)	Ischaemic cardiac arrest (%)	Unclassified death (%)	Total number of stroke	Haemorrhagic stroke (%)	Ischaemic stroke (%)	Unclassifiable stroke (%)
Gender									
Male	34053	5673	61.9	6.0	32.1	28380	24.0	72.6	3.4
Female	27226	4193	50.1	4.5	45.4	23033	20.0	76.2	3.8
Residence									
Urban	20524	4339	62.2	5.0	32.8	16185	24.5	70.8	4.7
Rural	40755	5527	52.6	5.7	41.7	35228	21.1	75.8	3.1
Age group									
<35	401	94	56.4	33.0	10.6	307	60.0	37.8	2.3
35–64	15994	1896	78.4	7.8	13.8	14098	29.4	68.0	2.6
> = 65	44884	7876	51.6	4.5	43.9	37008	19.1	76.9	4.0
Total	61279	9866	56.8	5.4	37.8	51413	22.2	74.2	3.6

The incidence, mortality and case-fatality rates of all acute CVD events were respectively 367.0 and 127.1 per 100,000, and 34.6% (Table 3). CHD had an incidence of 59.1 per 100,000, a mortality rate of 43.3 per 100,000 and a case-fatality rate of 73.2%. Regarding stroke, we observed the incidence of 307.9, mortality of 83.8 per 100,000, and a case-fatality rate of 27.2%. Compared with females, males had higher incidence and mortality, but slightly lower case-fatality rates for CHD, stroke and total acute CVD events (p<0.001). Compared with their rural peers, urban residents reported higher CHD incidence and mortality, but lower case-fatality rates (p<0.001). Regarding stroke and total acute CVD, urban residents reported lower incidence, but higher mortality and case-fatality rates than rural residents (p<0.001). Incidence and mortality of acute CVD events increased as age advanced.

**Table 3:** Crude and standardized† incidence (/100,000/year), mortality (/100,000/year) and case-fatality rates (%) of acute cardiovascular diseases by gender, residence and age groups in Zhejiang, China, 2012.

	All acute cardiovascular diseases			Coronary heart disease			Stroke		
	Crude and (standardized) incidence	Crude and (standardized) mortality	Crude and (standardized) case-fatality rate	Crude and (standardized) incidence	Crude and (standardized) mortality	Crude and (standardized) case-fatality rate	Crude and (standardized) incidence	Crude and (standardized) mortality	Crude and (standardized) case-fatality rate
Gender^a^									
Male	403.1 *** (290.9***)	134.2*** (97.3***)	33.30%*** (23.0%*)	67.1*** (49.0***)	45.5*** (33.3***)	67.8%*** (53.3%)	336.0*** (241.9***)	88.7*** (64.0***)	26.4%*** (13.4%)
Female	329.9 (213.2)	119.7 (70.8)	36.28% (16.5%)	50.9 (30.6)	41.7 (24.0)	80.5% (50.0%)	279.1 (182.6)	78.7 (46.8)	28.2% (12.0%)
Residence									
Urban	349.5*** (232.9***)	138.6*** (88.5***)	39.67%*** (19.6%)	73.9*** (48.2***)	50.9*** (32.5***)	68.8%*** (48.2%)	275.6*** (184.7***)	87.7*** (56.0)	31.8%*** (12.0%)
Rural	376.5 (260.5)	120.8 (80.8)	32.08% (21.7%)	51.1 (34.7)	39.1 (26.3)	76.6% (55.4%)	325.4 (225.8)	81.6 (54.5)	25.1% (13.4%)
Age group									
<35	5.7	1.1	20.4%	1.3	0.7	52.1%	4.3	0.5	10.7%
35–64	206.8	34.9	16.9%	24.5	12.5	51.2%	182.3	22.4	12.2%
> = 65	2367.7	972.5	41.1%	415.4	327.4	78.8%	1952.2	645.0	33.0%
Total	367.0 (250.8)	127.1 (83.7)	34.6% (20.2%)	59.1 (39.6)	43.3 (28.5)	73.2% (42.8%)	307.9 (211.2)	83.8 (55.1)	27.2% (14.3%)

***P<0.001;

* P<0.05

^†^ Standardized using the WHO Standard Population (2000–2025).

^a^ z test applied to examine differences in regarding incidence, mortality and case-facility between genders, as well as between residence status.

Table 4 shows that age and gender distributed evenly between the urban and rural areas in Zhejiang (p>0.05). No significant differences regarding all the rates were found between urban and rural areas among those aged below 35 years old (Table 5). For those aged 35 years and older, urban residents had higher CHD incidence, but lower stroke incidence (P<0.001) compared with rural residents. In addition, urban residents reported higher mortality for CHD and stroke than rural residents (P<0.001). However, rural residents had higher case-fatality rates on CHD compared with urban peers (P<0.01).

**Table 4:** Characteristics of urban and rural residence (in millions) in Zhejiang, China, 2012.

	Urban	Rural	Total
Total population	5.9	10.8	16.7
Age group, n (%)^a^			
<35 years	2.5 (42.4)	4.6 (42.6)	7.1 (42.5)
35–64	2.7 (45.8)	5.0 (46.3)	7.7 (46.1)
> = 65 years	0.7 (11.9)	1.2 (11.1)	1.9 (11.4)
Gender, n (%)^a^			
Male	3.0 (50.8)	5.5 (50.9)	8.5 (50.9)
Female	2.9 (49.2)	5.3 (49.1)	8.2 (49.1)

^a^ Pearson chi-square test was used to compare the population of urban areas with rural areas regarding age and gender. No significant difference was found (p>0.05).

**Table 5:** Difference in urban and rural residence in crude incidence (/100,000/year), mortality (/100,000/year) and case-fatality rates (%) by age, gender for acute cardiovascular disease subtypes in Zhejiang, China, 2012.

	Male						Female					
	Crude incidence		Crude mortality		Crude Case-fatality rate		Crude incidence		Crude mortality		Crude Case-fatality rate	
Age group	CHD†	Stroke	CHD	Stroke	CHD	Stroke	CHD	Stroke	CHD	Stroke	CHD	Stroke
<35^a^												
Urban	2.3	6.0	1.0	0.5	44.8%	9.2%	0.3	3.2	0.2	0.2	50.0%^b^	7.5%
Rural	1.9	5.3	1.2	0.6	60.9%	11.2%	0.7	3.0	0.3	0.4	40.0%	13.6%
35–64 ^a^												
Urban	45.8 ***	197.9 ***	19.7	29.5	42.9% ***	14.9%	14.7 ***	109.7 ***	7.6	13.0	51.8% **	11.8% ***
Rural	32.8	224.5	17.5	31.0	53.3%	13.8%	9.7	169.9	6.2	14.7	64.3%	8.6%
> = 65 ^a^												
Urban	560.0 ***	1947.8 ***	392.6 ***	683.0	70.1% ***	35.1% ***	475.0 ***	1588.6 ***	379.4 ***	670.6 ***	79.9% ***	42.2% ***
Rural	385.4	2246.6	301.9	678.3	78.3%	30.2%	335.0	1874.9	288.7	578.9	86.1%	30.9%

** p<0.01;

*** p<0.001.

^†^ CHD, coronary heart disease.

^a^ z tests applied to examine differences in regarding incidence, mortality and case-facility between genders, as well as between residence status.

^b^ 2×2 table exact test.

## Discussion

To our knowledge, this is the first study in China reporting population-based, real-time surveillance data on acute CVD events. ICDSS achieved a high level of completeness in reporting as the surveillance system obtained data directly from various sources. It also maintained a high level of validity and accuracy through ongoing and systematic quality assurance measures conducted in hospitals and CDCs. The procedures of ICDSS is similar to that of the US National Cardiovascular Disease Surveillance System [11] and the Cardio-cerebrovascular Disease Registration System in Takashima county, Japan [21].

The incidence, mortality and case-fatality rates of total acute CVD events were respectively 367.0 (CHD 59.1, stroke 307.9), 127.1 per 100,000 (CHD 43.3, stroke 83.8) and 34.6% (CHD 73.2%, stroke 27.2%) in Zhejiang in 2012. Mortality of CHD and stroke in Zhejiang are lower than China’s estimated national average (CHD, urban 86.3, rural 69.2 per 100,000; stroke, urban 125.1, rural 145.7 per 100,000) [7]. China does not have available data on national CVD incidence to be compared with [5]. The National CDC has started collecting CVD incidents from 302 counties since 2014, but robust data can only be reported after the system has been operated after several years. At the regional level, stroke incidence in our study was lower than that reported in the northeast provinces (i.e., 486 per 100,000) [22]. Our results were consistent with other studies that reported lower CVD incidence in south compared with the north in China [7, 19, 23]. These regional divergences may be related to China’s huge north-south variations in environmental, socioeconomic and behavioral factors. Zhejiang’s case-fatality rates of CHD and stroke were higher than urban districts of Beijing (53.6% and 17.3%) [24, 25].

Zhejiang had lower mortality for CHD but higher mortality for stroke compared with US and UK populations (standardized mortality rate of CHD: US 80.5, UK 68.8 per 100,000; stroke: US 25.4, UK 36.9 per 100,000) [2]. In addition, Zhejiang had lower incidence for CHD [26, 27], but higher incidence for stroke [28, 29] compared with UK and US. One plausible explanation may be that compared with western countries, China had lower prevalence of blood lipid abnormalities [30], the most important risk factors for CHD [31–33]. On the other hand, China had higher hypertension prevalence [22, 34], and higher smoking rates [35–37]. The case-fatality rates of CHD and stroke were higher in Zhejiang than those of western countries [34, 38]. This may be explained by China’s lack of stroke first-aid services, timely access to hospitals and adequate health insurance coverage for emergencies and inpatient services [16, 39].

In Zhejiang, CHD incidence and mortality were higher in urban areas than rural areas, which is probably due to the relatively higher prevalence of lipid abnormalities in urban areas [7]. We found that those aged 65 years or older presented the largest difference regarding CHD incidence and mortality between urban and rural areas. Currently in China, lipid profile examination is provided free for rural areas, but not for urban areas [40]. Our study suggested including lipid profile in the annual physical examination for urban residents. On the other hand, we found urban areas had a significantly lower CHD case-fatality rate than rural areas. In addition, we also found urban residents had lower stroke incidence, which echoed other studies [7, 41]. This may be due to a higher prevalence of hypertension and smoking in rural areas [42–45]. Furthermore, we found that the largest differences were found among the elderly. All these results implied that stroke prevention and management in rural areas was even more urgently needed compared with rural areas. Further research will be needed to explore why in our study the stroke fatality in the urban area was higher than that in the rural areas in Zhejiang, as other studies report otherwise in both China and elsewhere [7, 41, 46].

### Limitations

The study had several limitations. First, this study only included cross-sectional findings on acute CVD events in 2012. Further research on the temporal trend of the patterns of acute CVD over a long period will provide better evidence regarding epidemic change and policy implications. Second, as China displays a huge geographical diversity in CVD epidemiological characteristics, our findings based on one province may not be generalizable to the entire nation. Third, our urban-rural classification was based on the nature of the clusters, and not the household registration status of individuals in the clusters. This may introduce some misclassification errors, although our methods were consistent with definitions of the Sino-MONICA project [19] and the national myocardial infarction registry [13].

## Conclusions

Based on a relatively complete and reliable CVD surveillance system, we found that incidence and mortality of acute CVD were lower in Zhejiang province compared with the national average. Within the province, we found a significant urban and rural difference. Special attentions need to be given to stroke control, especially in rural areas.
